# Supplementary material for: Microbial Fertilization Improves Soil Health When Compared to Chemical Fumigation in Sweet Lily
Source: J Fungi (Basel). 2022 Aug 12;8(8):847. doi: 10.3390/jof8080847 (PMC9409919; doi:10.3390/jof8080847)
Supplement: Supplementary file 1 [file jof-08-00847-s001.zip › jof-1819425-supplementary.pdf]

Journal of Fungi

MDPI

Supplementary Materials

Microbial Fertilization Improves Soil Health When Compared to Chemical Fumigation in Sweet Lily

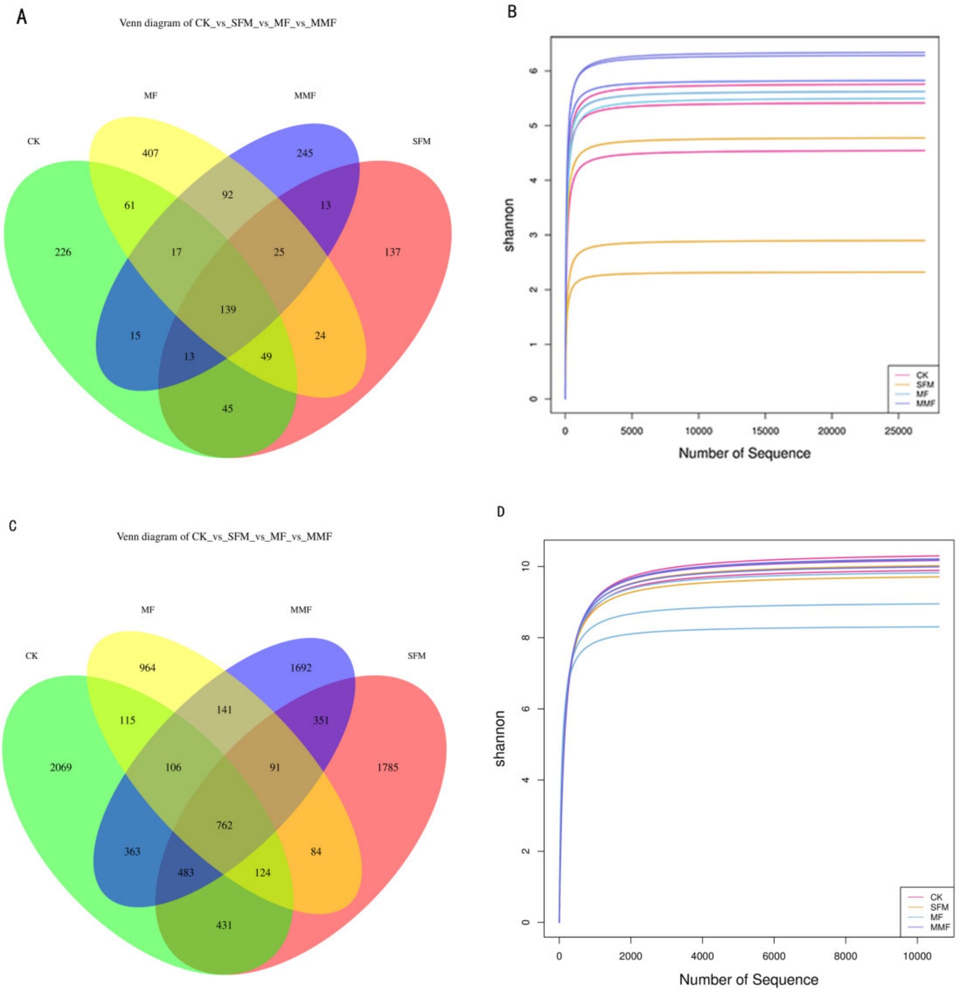

Supplementary Figure S2. Venn diagram of fungal OTUs (A) and Shannon-Wiener curves (B) and Venn diagram of bacterial OTUs (C) and Shannon-Wiener curves (D) of Lanzhou lily soil microorganism communities from Illumina NovaSeq data.

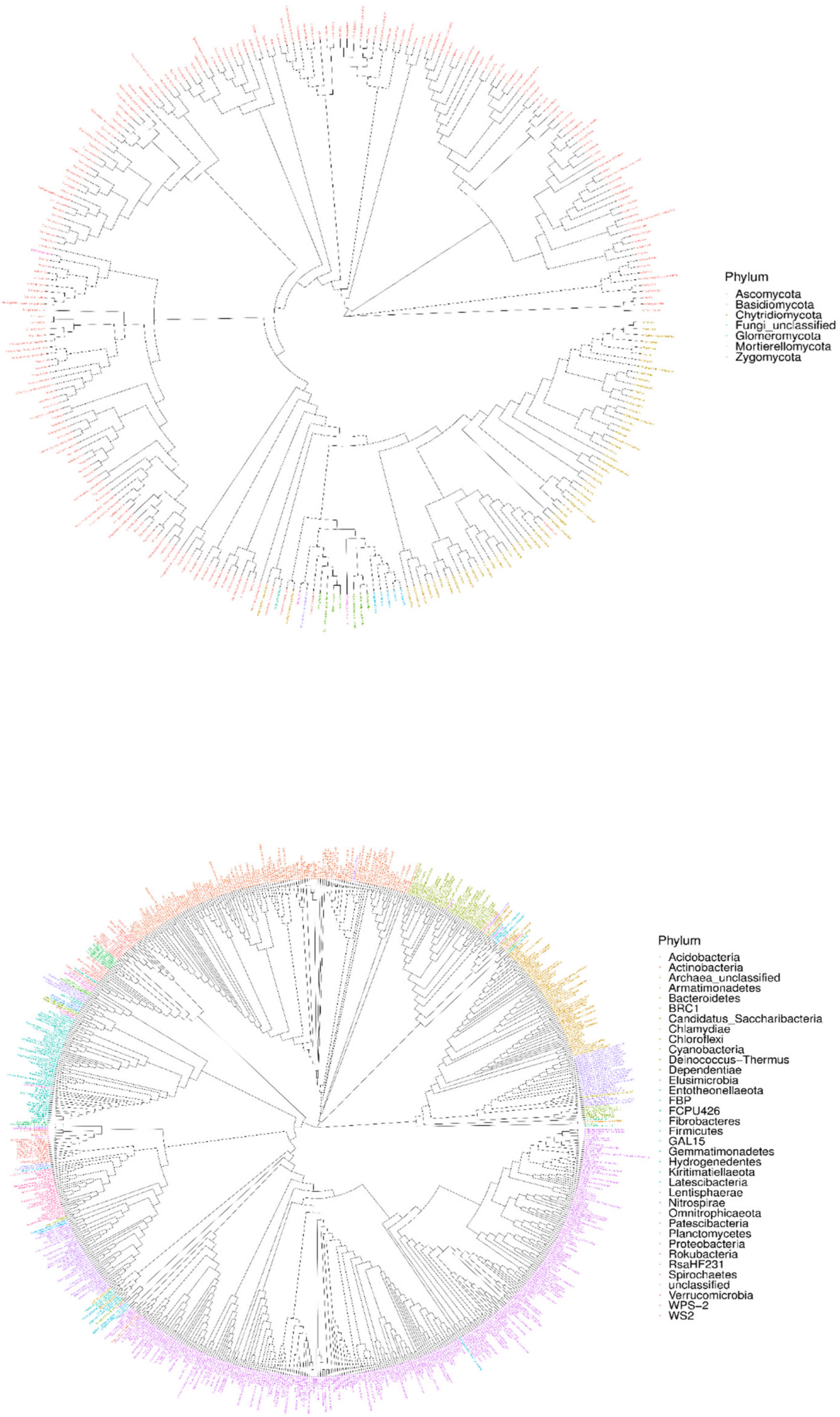

Supplementary Figure S2. The phylogeny tree of fungi (A) and bacteria (B) of Lanzhou lily rhizosphere soil among different treatments from Illumina NovaSeq.

Supplementary Table S1. *Sphingomonas* species and *Fusarium* species distribution of total reads of Lanzhou lily rhizosphere soil from Illumina NovaSeq data.

**Treatments**	**CK**	**SFM**	**MF**	**MMF**
(a) *Sphingomonas* species				
s__uncultured_Sphingomonas_sp.	1.23%	1.37%	2.82%	3.12%
s__Sphingomonas_unclassified	0.33%	0.45%	1.08%	1.14%
s__Sphingomonas_sediminicola	0.08%	0.17%	0.35%	0.21%
s__Sphingomonas_sp._CB_286425	0.03%	0.05%	0.03%	0.05%
s__Sphingomonas_lutea	0.00%	0.02%	0.05%	0.03%
s__Sphingomonas_sp._CA25	0.01%	0.00%	0.04%	0.04%
s__Sphingomonas_sp._CB_286428	0.00%	0.03%	0.00%	0.04%
s__Sphingomonas_sp._CB_286429	0.01%	0.00%	0.00%	0.04%
s__Sphingomonas_haloaromaticamans	0.00%	0.00%	0.00%	0.01%
(b) *Fusarium* species				
s__Fusarium_domesticum	1.87%	0.58%	1.50%	1.01%
s__Fusarium_merismoides	0.20%	0.34%	0.19%	1.22%
s__Fusarium_oxysporum_f_sp_psidii	0.00%	0.24%	0.00%	0.08%
s__Fusarium_solani	0.03%	0.03%	0.10%	0.05%
s__Fusarium_lateritium	0.07%	0.00%	0.00%	0.11%
s__Fusarium_redolens	0.04%	0.01%	0.01%	0.00%
s__Fusarium_tricinctum	0.01%	0.03%	0.00%	0.01%
s__Fusarium_lunatum	0.00%	0.00%	0.00%	0.03%
s__Fusarium_cuneirostrum	0.01%	0.00%	0.00%	0.00%

Supplementary Table S2. Summary of soil physicochemical properties of Lanzhou lily rhizosphere soil among different treatments: the data has published [16].

**Treatment**	**Bulk density (g · cm-3)**	**Moisture content (%)**	**pH**	**Salt content (g · kg-1)**	**Organic matter (g · kg-1)**	**Available nitrogen (mg · kg-1)**	**Available phosphorus (mg · kg-1)**	**Available potassium (mg · kg-1)**
CK	1.33±0.01a	14.63±0.09a	7.76±0.03c	0.48±0.01d	13.21±0.10d	65.16±1.01c	94.48±0.93d	236.55±2.8a
SFM	1.32±0.01ab	14.20±0.21b	7.87±0.03b	0.58±0.01c	13.95±0.13c	71.29±0.86b	146.18±5.98c	219.71±3.54b
MF	1.31±0.01b	13.19±0.11c	7.90±0.03ab	0.80±0.01b	14.74±0.13b	82.57±0.99a	194.33±2.59b	204.89±3.69c
MMF	1.30±0.01b	12.09±0.07d	7.99±0.03a	1.08±0.04a	16.18±0.38a	83.25±0.80a	209.95±3.35a	171.04±3.25d
